# NGAL and HPV Subtypes in Cervical Carcinoma: Implications for Cancer Progression and Treatment Response

**DOI:** 10.3390/cimb48020234

**Published:** 2026-02-23

**Authors:** Behar Raci, Snezana Stojkovska, Gezim Hodolli, Violeta Klisarovska, Goran Dimitrov, Shemsi Veseli, Arta Kameri-Jusufi, Mentor Kurshumliu, Diellor Rizaj, Arben Sinani

**Affiliations:** 1 Clinic of Oncology, University Clinical Center of Kosovo, 10000 Prishtina, Kosovo; behar.raci12@gmail.com (B.R.); artakameri1980@gmail.com (A.K.-J.); 2 Faculty of Medicine, University Clinic for Infectious Diseases & Febrile Conditions, 1000 Skopje, North Macedonia; snezanast@gmail.com; 3 Faculty of Veterinary and Agriculture, University of Prishtina, 10000 Prishtina, Kosovo; gezim.hodolli@uni-pr.edu; 4 Faculty of Medicine, University Clinic of Radiotherapy and Oncology, 1000 Skopje, North Macedonia; violeta.klisarovska@medf.ukim.edu.mk; 5 Faculty of Medicine, University Clinic of Gynecology and Obstetrics, 1000 Skopje, North Macedonia; gorandimi@gmail.com; 6 Biochemistry Clinic, University Clinical Center of Kosovo, 10000 Prishtina, Kosovo; shemsi.veseli@gmail.com; 7 College of Medical Sciences Heimerer, 10000 Prishtina, Kosovo; mentor@prolab-ks.com; 8 Clinical Rheumatology, University Clinical Center of Kosovo, 10000 Prishtina, Kosovo; diellor.rizaj@ubt-uni.net

**Keywords:** NGAL, HPV, cervical cancer, biomarkers, inflammation

## Abstract

**Background/Objectives**: Cervical cancer is a prominent source of morbidity and mortality among women, particularly in low- and middle-income nations. Neutrophil Gelatinase-Associated Lipocalin (NGAL), a glycoprotein involved in cancer-related activities, has been proposed as a biomarker; however, its involvement in cervical cancer remains unknown. The study aim is to evaluate the prognostic significance of serum NGAL levels in cervical cancer patients in relation to International Federation of Gynecology and Obstetrics (FIGO) stage, operability, and HPV subtype distribution before and after treatment. **Methods**: The study involved 130 women, 100 with histologically proven cervical cancer and 30 healthy controls. The serum NGAL levels were determined before and after treatment using an ELISA test. HPV genotyping was carried out using real-time PCR on 21 high- and low-risk subtypes. **Results**: NGAL levels increased marginally during therapy (from 134 to 144 ng/mL; *p* = 0.28), but the rise was significant in inoperable patients (*p* = 0.02) and increased with advanced FIGO stage, although this did not reach statistical significance (*p* = 0.07). HPV 16 was the most common subtype (26.0%), while women aged 51–60 had the highest overall HPV positive rate (72.7%). There was no significant association between NGAL levels and HPV subtypes (*p* = 0.17). **Conclusion**: NGAL does not appear to be an accurate short-term indicator of therapy response. However, increased levels in advanced-stage and inoperable instances indicate prognostic significance. NGAL most likely represents tumor-associated inflammation rather than HPV subtype. These findings support its possible inclusion in future biomarker panels, subject to validation in bigger investigations. Persistent HPV infection in midlife women highlights the significance of ongoing screening.

## 1. Introduction

Cervical cancer places a significant burden on women’s health, especially in low- and middle-income countries where access to preventive screening and early treatment is limited. Globally, it is the fourth most common cancer among women, with an estimated 604,000 new cases and 342,000 deaths in 2020 [1]. Its pathogenesis is strongly linked to persistent infection with high-risk human papillomavirus (HPV) types, most notably HPV 16 and HPV 18, which together account for over 70% of cervical cancer cases [2,3]. While the function of HPV in beginning carcinogenesis is well recognized, the progression from infection to invasive cancer depends on host immunity, viral genotype, and molecular modifications within the tumor microenvironment [4,5].

In 2022, cervical cancer was expected to cause 662,300 new cases and approximately 348,900 deaths worldwide. Cervical cancer remains the fourth most frequent malignancy and the fourth largest cause of cancer-related deaths in women worldwide [6]. Approximately 94% of deaths occur in low- and middle-income countries (LMICs), emphasizing global health disparities [7]. Each year, cervical cancer causes around 46,000 new cases and 21,000 fatalities in Eastern Europe and the Balkans. In Romania (2019), cervical cancer had the largest burden in the region, with an incidence rate of 20.4 per 100,000 women, as well as high Disability-Adjusted Life Years (DALYs) and Years of Life Lost [8]. Some nations, such as Slovenia, Greece, and Serbia, saw decreases in incidence and death between 1990 and 2019, owing to enhanced screening and HPV vaccination programs.

According to recent data from Kosovo, the number of women diagnosed with reproductive organ malignancies—a group that includes cervical cancer—has climbed from 73 instances in 2012 to 361 cases in 2021 [9]. Furthermore, the study Management of Noncommunicable Diseases in Kosovo notes an increase in cervical cancer-related deaths, despite the fact that no official national statistics for recent years give age-standardized mortality rates (per 100,000 women) [10]. This lack of specific mortality data highlights the critical need for comprehensive national cancer registries to support strong epidemiological analysis and improve preventive and treatment programs.

Neutrophil Gelatinase-Associated Lipocalin (NGAL), also known as lipocalin-2, is an emerging molecular marker of relevance in cancer biology. NGAL is a 25 kDa glycoprotein that was originally identified as a component of neutrophil granules but is now known to be released by numerous epithelial and tumor cells in response to proinflammatory stimuli and tissue injury [11]. It is implicated in iron sequestration, immune response regulation, and extracellular matrix remodeling—all of which are closely linked to tumor formation and metastasis [12,13].

In recent years, the role of NGAL has been investigated across a variety of tumor types. Elevated NGAL levels have been documented in breast, pancreatic, endometrial, and colorectal malignancies, where they correlate with poor prognosis, invasiveness, and resistance to chemotherapy [11,12]. Mechanistically, NGAL has been found to induce epithelial–mesenchymal transition (EMT), a vital stage in tumor invasion and metastasis, and interact with matrix metalloproteinase-9 (MMP-9), boosting its enzymatic activity and aiding tissue remodeling [14]. Furthermore, NGAL may influence immune evasion, tumor-associated angiogenesis, and iron-dependent cell proliferation, which are all critical mechanisms in cancer progression [15].

However, in the context of cervical cancer, the clinical and biological ramifications of NGAL are little understood. Few studies have investigated its expression in cervical cancer tissues or its potential as a non-invasive biomarker. NGAL has been extensively researched in different malignancies such as breast, pancreatic, and colorectal cancer [11,12,16,17]. Its significance in cervical cancer is far less understood. Only a few studies have investigated NGAL expression in cervical cancer tissues or its potential as a circulating biomarker [15,18]. These studies imply that NGAL may play a role in tumor-related inflammation; however, they are preliminary and generally constrained by small sample sizes. As a result, significant research gaps remain about whether NGAL correlates with tumor stage, operability, or HPV subtype in cervical cancer. Our study fills this gap by examining blood NGAL levels before and after treatment, as well as FIGO staging, operability status, and HPV genotyping in a group of 130 patients, providing new insights into its predictive significance.

Although Dhankhar et al. [19] demonstrated that serum NGAL varies according to treatment duration and radiation modality in locally advanced cervical cancer, their study did not clarify whether NGAL primarily reflects treatment-related inflammation or intrinsic tumor biology, nor did it account for HPV status or tumor operability. Moreover, their cohort was limited to 30 patients with advanced disease and lacked healthy controls, early-stage tumors, and virological stratification.

In contrast, the present study extends this framework by integrating serum NGAL with FIGO staging, operability status, and HPV genotyping in a larger cohort of 100 cervical cancer patients and 30 healthy controls. This design allows us to disentangle treatment-related effects from biologically driven NGAL expressions and to evaluate whether NGAL reflects tumor aggressiveness, systemic inflammatory burden, or HPV-associated oncogenic activity. To our knowledge, no previous study has simultaneously analyzed NGAL dynamics in relation to operability, FIGO stage, and HPV subtype in cervical carcinoma. However, given the chronic inflammation and persistent oxidative stress associated with high-risk HPV infection, it is possible that NGAL plays a role in the tumorigenic cascade of cervical neoplasia [18]. Furthermore, because NGAL can be quantified in blood and urine, it is an appealing option for dynamic monitoring of disease load and therapy response.

The current study intends to investigate the relationship between NGAL levels and HPV subtype distribution in a cohort of cervical cancer patients. Specifically, we look into whether NGAL levels correlate with FIGO staging and whether there are differences between pre- and post-treatment values. Furthermore, we investigate HPV genotypes in connection to clinical staging and age, gaining a better knowledge of how viral and host inflammatory indicators may interact to shape disease development. By understanding these associations, we hope to assess NGAL’s value as a potential prognostic and predictive biomarker in cervical cancer, as well as to inform future tailored therapy options.

## 2. Materials and Methods

### 2.1. Study Design and Population

This study was designed as a prospective observational cohort conducted between 2023 and 2025 at the University Clinical Center of Kosovo (UCCK), Prishtine, Kosovo, and involved 130 women: 100 with histologically proven cervical cancer (ages 25–75) and 30 healthy controls (ages 20–60). The study received ethical approval from the Ethical Committee of the Chamber of Doctors of Kosovo (Approval No. 124/23, 14 July 2023). Written informed consent was obtained from all participants before enrollment in the study. No more blood samples were taken beyond what was required by routine procedure. Control subjects, drawn from the medical staff, were free of cancer, chronic illnesses, or inflammatory conditions. In clinical biomarker research, sample size is often set by the predicted prevalence of the condition in the target population, the effect size to be observed between groups, and the necessary statistical power (usually 80–90% when α = 0.05). The current study comprised 100 cervical cancer patients and 30 healthy controls (a total of 130), which is similar with regional biomarker studies that typically enroll 80–150 patients in single-center cohorts from Serbia, Albania, and Bosnia. Given that HPV prevalence in cervical cancer surpasses 70% in most Balkan series, this sample size has the power to distinguish between HPV genotypes, FIGO stage groups, and operability status.

Serum NGAL measurements were performed at two predefined time points. Baseline samples were obtained 3–5 days before initiation of any therapeutic intervention, including surgery, radiotherapy, or chemoradiotherapy, depending on disease stage. Follow-up samples were collected after completion of the entire treatment protocol. In patients treated with surgery or radiotherapy alone, post-treatment blood sampling was performed 2–4 weeks after therapy completion. In patients receiving concurrent chemoradiotherapy, follow-up samples were obtained 9–12 weeks after completion of combined treatment in order to minimize the influence of acute radiation- and chemotherapy-induced inflammatory responses on circulating NGAL levels. Eligible participants had histologically confirmed cervical cancer, with a healthy control group providing informed consent. Exclusion criteria included systemic or chronic diseases affecting biochemical or hematological parameters (diabetes, autoimmune disorders, hypertension, hyperlipidemia, viral infections), renal dysfunction (urea or creatinine > 2× upper limit), hepatic impairment (AST, ALT, bilirubin > 2× upper limit), and any acute cervical or systemic inflammatory condition at the time of blood sampling. In early-stage instances, such as FIGO stage IA, where brachytherapy alone was advised, NGAL sample was performed three days before brachytherapy began and again two weeks after treatment completed.

Stage (FIGO IA1-IB1) underwent two 9 Gy brachytherapy sessions (a total of 18 Gy). Those with FIGO IB1 or higher (up to IIIA without metastases) received EBRT using intensity-modulated radiation based on histological grade and risk factors. EBRT was administered at 1.8 Gy/day to 45 Gy, with a 5.3 Gy boost in three portions (total 50.4 Gy). The patient received weekly cisplatin (50 mg/m^2^) for five weeks. Blood and organ function were checked weekly. Clinical factors dictated that brachytherapy comes after EBRT.

### 2.2. Sample Collection and Processing

All blood samples were collected at the Oncology Clinic, University Clinical Center of Kosovo (UCCK), under standardized clinical conditions. A 5 mL venous blood sample was drawn from each participant into plain tubes, ensuring minimal pre-analytical variability. Of this, 2.6 mL was specifically allocated for NGAL analysis, while the remainder was used for parallel biochemical and hematological assessments. A control group consisting of 30 healthy individuals was also included to provide baseline NGAL values for comparative analysis. Strict procedural consistency was maintained: samples were drawn intravenously by trained medical personnel, using sterile technique, and processed without delay to prevent protein degradation. The plasma was centrifuged and kept at −70 °C for further biochemical analysis.

After collecting, all blood samples were allowed to coagulate and then centrifuged at 3000 rpm for 10 min to separate serum. The serum was then aliquoted, labeled, and processed using the ELISA kit procedure.

NGAL samples were diluted (≥2-fold) to optimize assay conditions and stay within the linear detection range. Each sample was tested in triplicate wells to assure accuracy, and the results were averaged across replicates to eliminate intra-assay variation. Optical density was measured at 450 nm using a microplate reader, and the findings were interpolated against a manufacturer-provided calibration curve. Every assay plate underwent stringent quality control procedures, including the use of blank wells, internal controls, and standard references to ensure reproducibility.

### 2.3. NGAL Quantification

The analytical methodology employs a sandwich ELISA kit (BioPorto Diagnostics A/S, Hellerup, Denmark; KIT-036) to quantify serum levels of Neutrophil Gelatinase-Associated Lipocalin (NGAL). The ELISA utilizes microwells pre-coated with a monoclonal capture antibody specific for human NGAL. After incubation with diluted serum samples alongside calibrators and controls, a biotinylated detection antibody is added, followed by horseradish peroxidase (HRP)-conjugated streptavidin. The enzymatic complex catalyzes a chromogenic reaction upon addition of a tetramethylbenzidine (TMB) substrate, yielding a color change that is measured spectrophotometrically at 450 nm. This assay demonstrates high analytical sensitivity, with a limit of detection as low as approximately 4 pg/mL and a linear dynamic range spanning from 10 to 1000 pg/mL. NGAL is an early and robust biomarker of tissue injury, particularly acute kidney injury, rising within hours—well before changes in serum creatinine—thus enabling timely clinical insight. To ensure data accuracy, all samples were assayed in duplicate, calibration curves were generated using kit-provided standards, and results were validated against established reference values to confirm both precision and reliability [16,17]

### 2.4. HPV Genotyping

HPV detection and genotyping were performed using the iPonatic S-Q31A&B system, version 3.2, with the HPV 13+2 Human Papilloma Virus DNA Diagnostic Kit (Sansure Biotech Inc., Changsha, China; CE-IVD). This real-time PCR-based device detects 21 high- and low-risk HPV subtypes and is CE-IVD-certified. The approach has proven great sensitivity and specificity in detecting numerous HPV strains related to cervical oncogenesis [20,21]. HPV genotyping was performed using the HPV Quant 21 real time PCR in vitro diagnostic assay, which detects 21 high and low risk HPV subtypes. The assay includes HPV types 6, 11, 16, 18, 26, 31, 33, 35, 39, 44, 45, 51, 52, 53, 56, 58, 59, 66, 68, 73, and 82. Genomic DNA was extracted from cervical specimens using the DNA extraction protocol provided by the manufacturer, according to standardized PCR-based molecular diagnostic procedures. All analyses were conducted in accordance with the manufacturer’s instructions. HPV testing provided qualitative results (positive/negative) and genotype identification only; viral load quantification was not performed.

All laboratory studies were completed at University Clinical Center of Kosovo (UCCK) certified by ISO-compliant quality control techniques. The hematology analyzers (Horiba Yumizen H500, Kyoto, Japan) were used to measure the complete blood count (CBC). The leukocytes and hemogram values, as well as biochemical parameters (urea, creatinine, AST, ALT, and bilirubin) were measured with Horiba Pentra C400 (Kyoto, Japan).

### 2.5. Approach to Diagnosis of CA CX

In early-stage cancer, ranging from FIGO stage IA1 to IIA-IIB, the diagnosis was primarily made through surgical intervention. The majority of these individuals underwent Wertheim radical hysterectomy. The excised surgical specimens were then sent to the Institute of Pathology for a final diagnosis, which included a thorough histological examination and reporting of all pertinent parameters.

In advanced cases, defined as FIGO stage IIB, IIIA, or higher, where surgical therapy was not possible, the diagnosis was made through histological confirmation via biopsy sampling. Additional diagnostic methods included Papanicolaou (PAP) testing, imaging modalities such as pelvic and abdominal MRI, endometrial curettage, laboratory assessment of tumor markers (CEA and CA-125), and comprehensive biochemical profiling.

## 3. Results

### 3.1. NGAL Levels Before and After Treatment

**Figure 1:**
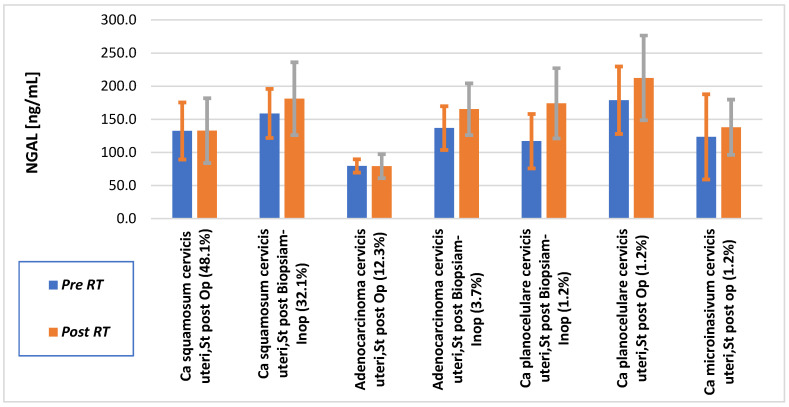
Average NGAL levels before and after radiotherapy by histopathological findings. Aberrations are staying for: Ca—carcinoma, St—Status, Op—Operable, Inop—Inoperable. Mean values are presented as bars, and the orange and gray error bars denote the standard deviation for the pre- and post-treatment measurements, respectively.

The paired study of NGAL levels before and after chemoradiation (CH-RT) across several cervical cancer subtypes revealed distinct patterns based on histology and operability status (Figure 1).

For patients with Ca squamosum cervicis uteri operable patients, the mean NGAL level did not change, it was 132 ng/mL pre-RT to 132 ng/mL post-RT, but this difference was not statistically significant (*p* = 0.61), indicating that there is no meaningful treatment effect on NGAL expression in operable squamous cell carcinoma. In contrast, for Ca squamosum cervicis uteri inoperable, the NGAL level increased from 158 ng/mL to 181 ng/mL, representing a 22.3 ng/mL increase following biopsy. Although this rising trend suggests a link between tumor inoperability and aggressiveness, the difference was not statistically significant (*p* = 0.35).

NGAL levels in adenocarcinoma cervicis uteri were constant after surgery (79.5 to 79.3 ng/mL; *p* = 0.98). In inoperable cases, NGAL increased (from 136 to 165 ng/mL; *p* = 0.60), which was most likely limited by the small sample number. Rare subtypes, such as inoperable planocellular and microinvasive carcinoma, demonstrated post-treatment NGAL increases but were not statistically evaluated because of low case numbers.

### 3.2. Analysis by Clinical Stage

To evaluate the link between disease stage and inflammatory activity, we compared NGAL levels before and after treatment, stratified by FIGO classification. The average pre- and post-treatment NGAL levels across FIGO phases are summarized in Table 1. For comparative purposes, NGAL was also evaluated in 30 healthy individuals who comprised the control group. In this group (*n* = 30), the mean NGAL level was 66.1 ng/mL. The values ranged from 39.9 to 116 ng/mL, with a median of 66.9 ng/mL. The interquartile range (IQR) was 54.8 to 73.2 ng/mL. Increased NGAL levels were observed across most cervical cancer subtypes. However, no elevation was detected in cases of operable adenocarcinoma of the cervix uteri.

In terms of initial tumor size, patients with tumors smaller than 4.5 cm (*n* = 54) had a diverse distribution, with lower mean NGAL values of 108 and 122 ng/mL for Pre and Post treatment, and 75.9% of cases for Concomitance Chemoradiotherapy (includes External an Internal radio treatment) (CCRT) and 24.1% of cases for Brachytherapy (BRT). On the other hand, patients with tumors larger than 4.5 cm (*n* = 46) showed a homogenous distribution, with all cases (100%) fitting into a single category and significantly higher mean values (178 and 175 ng/mL) before and after therapy.

In terms of clinical nodal involvement, patients without nodal involvement (*n* = 44) showed a mixed distribution, with lower mean values of 101 and 107 ng/mL of NGAL for pre and post treatment, with 70.5% belonging to CCRT and 29.5% to BRT. On the other hand, individuals with clinically positive nodes (*n* = 56) had a higher mean value of 157 ng/mL and complete clustering in one category (100%). These results suggest that higher values of the assessed parameters are linked to bigger initial tumor size and the presence of clinical nodal involvement.

NGAL levels indicated no significant (*p* > 0.31) pre/post-treatment variations in the early stages (IA-IB), with FIGO IIB being the only stage with a statistically significant increase.

The lack of significant NGAL changes in early-stage disease (IA–IB) shows that in confined tumors with a lower inflammatory burden, NGAL may not be a sensitive short-term response measure. This stability is consistent with the good prognosis of early-stage cervical cancer, where systemic inflammation is low.

Although FIGO stage IIB showed the largest numerical increase in NGAL levels, this trend did not consistently reach statistical significance and should therefore be interpreted as a biological signal rather than a confirmed prognostic threshold.

In advanced phases (IIIA, IIIC), NGAL values remained high but did not change statistically significantly. This could indicate tumor heterogeneity, necrosis, or a small sample size. Nonetheless, repeatedly increased levels indicate a high inflammatory environment, even if not statistically significant (*p* = 0.15).

Overall, NGAL appears to be a restricted short-term dynamic marker in early stages, but it has increased prognostic relevance in intermediate to advanced disease, notably in FIGO IIB, where its rise may serve as an early warning signal of physiologically aggressive disease.

### 3.3. NGAL Levels in Operable vs. Inoperable Patients

Patients were classified as operable or inoperable according to their histological findings and TNM status. Table 2 shows the average NGAL levels before and post treatment for both inoperable and operable patients.

Statistical analysis using independent *t*-tests revealed that pre-treatment NGAL was greater in inoperable patients, but the difference approached significance: t = 1.93, *p* = 0.06; and post-treatment NGAL was significantly higher in inoperable patients: t = 2.37, *p* = 0.02. These findings indicate that inoperable patients have a substantially greater inflammatory or tumor-related NGAL response following treatment, as well as a clear pattern of elevated NGAL even prior to therapy.

### 3.4. HPV Genotype Distribution and NGAL

HPV subtypes varied throughout the sample, with a prevalence of high-risk genotypes (HPV 16, 18, 31, and 52). There was no statistically significant (*p* = 0.39) correlation between HPV subtypes and NGAL expression levels, either before or after treatment (Kruskal–Wallis test).

Although NGAL levels increased following treatment in several patients, the difference was not statistically significant (*p* = 0.28). There was no significant (*p* = 0.41) association between NGAL values and age, FIGO stage, operability, or HPV genotype, implying that NGAL may not be a short-term treatment response biomarker but may still be relevant in understanding long-term disease biology.

### 3.5. Analysis of HPV Subtypes

HPV-positive cases were grouped according to oncogenic risk, with HPV 16 and 18 examined individually, including co-infection. Other high-risk kinds were classified as “HPV_Other_HighRisk,” and no low-risk variants were found. HPV 16 had the highest prevalence (26.03%), followed by HPV 16/18 co-infection (8.22%) and HPV 18 alone (2.74%). Other high-risk kinds accounted for 24.6%. Overall, 63.0% were HPV positive (Table 3). These findings emphasize the prevalence of high-risk types, particularly HPV 16, and recommend subtype-specific analysis for clinical assessment.

To determine HPV prevalence, patients were divided into age groups. HPV positive rose with age, peaking between 51 and 60 years, and subsequently declined in individuals above 60. This shows that chronic or reactivated infections are more common in midlife, probably due to immunosenescence. The decrease in older women may be due to lower detection rates or testing bias.

The findings highlight the necessity of ongoing HPV surveillance beyond reproductive age, particularly for people in mid- to late adulthood, where persistent high-risk HPV infections may have a higher carcinogenic potential. According to the research, women under 30 have the greatest HPV positive (100%), followed by those aged 51–60 (72.7%). A decrease is noted in women over 60 (36%), implying age-related changes in infection persistence or detection, with midlife and younger women having the highest infection rates.

### 3.6. Statistical Significance

NGAL levels exhibited a biological pattern of increasing with FIGO stage, as is shown in Table 1, with significantly lower values in early-stage disease (e.g., FIGO IA1 pre-treatment = 105 ng/mL) compared to advanced disease (e.g., FIGO IIIA pre-treatment = 205 ng/mL). However, these changes were not statistically significant (pre-treatment ANOVA *p* = 0.23; post-treatment *p* = 0.07), highlighting the need for larger cohorts to validate stage-related associations.

Within-group analysis (Table 2) showed a significant increase in NGAL levels in inoperable patients (155 vs. 179 ng/mL, *p* = 0.04), whereas no significant change was observed in operable patients (122 vs. 123 ng/mL, *p* = 0.06). Between-group comparisons demonstrated higher NGAL levels in inoperable patients both before treatment (155 vs. 122 ng/mL, *p* = 0.06, trend toward significance) and after treatment (179 vs. 123 ng/mL, *p* = 0.02). This lends support to the theory that NGAL reflects systemic inflammatory burden and tumor aggressiveness in non-respectable illnesses.

HPV subtype analysis confirmed the prevalence of high-risk genotypes (HPV16 = 26.0%, HPV16 and 18 = 8.2%, and HPV18 = 2.7%), with no low-risk types found. There was no significant (*p* = 0.37) link seen between HPV subtype and NGAL levels (Kruskal–Wallis), indicating that NGAL elevation is independent of viral genotype. Taken together, our findings show that NGAL may serve as an indication of tumor aggressiveness and systemic inflammatory burden, particularly in advanced and incurable cervical malignancies, although validation in larger prospective cohorts is required

## 4. Discussion

NGAL has been widely investigated in several malignancies, including breast, colorectal, pancreatic, and endometrial cancers, where it has been associated with tumor aggressiveness, invasiveness, and treatment resistance. In cervical cancer, currently established biomarkers such as HPV subtyping, p16, and Ki-67 are mainly used for diagnosis and assessment of HPV-driven oncogenic activity but do not capture systemic tumor-associated inflammation or treatment-related biological responses. In contrast, serum NGAL provides a minimally invasive biomarker reflecting host inflammatory and tumor–microenvironment interactions, which may complement existing tissue-based markers in monitoring disease progression and treatment response.

The most consistent finding of this study was the higher serum NGAL levels observed in inoperable patients compared with operable cases, with statistical significance after treatment, supporting an association between elevated NGAL and tumor inoperability, whereas no association was observed between NGAL and HPV subtype. This pattern indicates that NGAL reflects tumor-associated systemic inflammation and tissue remodeling rather than viral genotype or short-term treatment response. These two observations form the central biological message of our study and provide a framework for interpreting the stage and histology related trends observed in subsequent analyses.

### 4.1. NGAL as a Biomarker of Treatment Response

In this study, NGAL levels showed no significant increase after treatment (mean change from 134 to 144 ng/mL; *p* = 0.28). Similar trends have been found in other malignancies where NGAL levels are still increased due to post-treatment inflammation or tissue remodeling rather than direct tumor shrinkage [17]. Neutrophil activity and matrix degradation modulate NGAL expression, which may continue to be active throughout early post-radiotherapy intervals. As a result, when interpreting NGAL during follow-up, it is important to consider its delayed normalization due to host inflammatory response rather than thinking it reflects real-time tumor dynamics. The slight increase in serum NGAL observed after treatment is biologically plausible and likely reflects radiation- and chemotherapy-induced tissue injury rather than tumor progression. NGAL is actively produced by activated neutrophils, macrophages, and damaged epithelial cells and is upregulated during oxidative stress, extracellular matrix remodeling, and wound-healing responses. Chemoradiation induces acute inflammatory cascades and stromal activation, which may sustain or even elevate circulating NGAL levels despite tumor regression. This explains why NGAL does not behave as a short-term treatment response marker but rather reflects the balance between tumor burden and host inflammatory activity.

### 4.2. NGAL Levels in Relation to Disease Stage

Although NGAL levels were higher in advanced FIGO stages (IIB, IIIA, and IIIC), no statistical significance was found (*p* = 0.23 pre-treatment; *p* = 0.07 post-treatment). NGAL levels generally rose as FIGO stage progressed, especially from early (IA-IB) to more advanced disease (IIB and IIIA). However, the phrase did not adhere to a tight linear pattern. FIGO IIIC showed a reduction compared to FIGO IIIA (302 ng/mL vs. 208 ng/mL). This non-linear trend implies that NGAL is more than just a stage-progression marker; it represents the intricate interplay of tumor biology, inflammatory response, and treatment-related alterations.

The observed drop in IIIC could be attributable to numerous factors: Tumor heterogeneity in mature stages, where necrosis and decreased viable tumor bulk can influence biomarker secretion. Prolonged inflammation, immunological fatigue, and treatment-induced alterations can all have an effect on circulating NGAL levels, the similar results were published by Kahraman et al. [22].

The near-significant post-treatment *p*-value suggests a biological trend, which could be related to hypoxia, necrosis, or persistent inflammatory responses that are more common in advanced tumors. Previous research has shown that NGAL promotes tumor progression through processes such as iron trafficking, epithelial–mesenchymal transition (EMT), and MMP-9 stabilization, which are more active in aggressive tumors [11,23]. The observed trend emphasizes NGAL’s potential prognostic value in staging; however, validation in larger cohorts is required.

### 4.3. NGAL and Operability Status

According to FIGO staging, operable cervical cancer cases are primarily in the early stages (IA1-IB1 and selected IIA), where surgical resection, often radical hysterectomy with pelvic lymphadenectomy, is possible. Inoperable cases are typically advanced stages (≥IIB) with parametrial invasion, pelvic sidewall extension, or distant spread, requiring concurrent chemoradiotherapy and brachytherapy. In the current cohort, operable patients in early stages had stable NGAL values before and after treatment (122 → 123 ng/mL), while inoperable patients in advanced stages had significantly higher NGAL levels pre-treatment (155 ng/mL) and post-treatment (179 ng/mL), with statistical significance after therapy (*p* = 0.02). Mean NGAL levels support this pattern, ranging from 98.3 ng/mL in FIGO IA1 to 294 ng/mL in FIGO IIIA, and exceeding 300 ng/mL post-therapy in FIGO IIB. These findings are consistent with clinical reality, where NGAL increase is associated with advanced stage and inoperability, implying its potential as a biological biomarker of tumor aggressiveness and systemic inflammatory burden.

The findings demonstrated that inoperable patients had consistently higher NGAL levels than operable patients, both before (155 vs. 122 ng/mL, *p* = 0.05) and after therapy (179 vs. 123 ng/mL, *p* = 0.02). While this may suggest a link to tumor aggressiveness, a deeper look reveals that NGAL increase is more likely to reflect inflammatory and tissue-remodeling processes associated with advanced disease than aggressiveness in a strict oncological sense.

NGAL overexpression is biologically consistent with neutrophil activation, extracellular matrix remodeling, and systemic inflammation, all of which are generally increased in bulky or inoperable tumors. This explanation also explains the lack of a linear trend across FIGO stages (e.g., lower levels in IIIC vs. IIIA) and the lack of correlation with HPV subtype pathogenicity.

Thus, NGAL should not be seen as a stage- or HPV-specific marker, but rather as a measure of host inflammatory burden and tumor microenvironmental alterations. In this position, NGAL may act as a predictive biomarker, particularly for individuals with a high inflammatory tumor load.

The operability was defined using FIGO staging and clinical criteria. Operable cases related to early stages (IA, IB1, and selected IIA) in which radical surgery was possible. Advanced instances (≥IIB) with parametrial invasion, pelvic wall involvement, or patient comorbidities necessitated chemoradiotherapy over surgery. NGAL levels followed this classification, with considerably higher values in inoperable patients after treatment (179 vs. 123 ng/mL, *p* = 0.02), supporting the idea that operability status is biologically connected to systemic tumor aggressiveness.

### 4.4. Histopathological Correlation of NGAL

This study examined NGAL alterations before and after treatment in several cervical cancer subtypes. In operable squamous carcinoma, NGAL levels were constant (132 to 132 ng/mL; *p* = 0.60). It increased noticeably in inoperable instances (158 to 181 ng/mL), but not substantially (*p* = 0.35). Rare subtypes, such as inoperable planocellular carcinoma, demonstrated significant increases (+57.1 ng/mL), although sample quantities were insufficient for statistical analysis. Overall, higher NGAL levels after therapy appear to be associated with inoperability and aggressiveness, indicating its potential as a prognostic marker pending further investigation [18].

### 4.5. Relationship Between HPV Subtypes and NGAL

Despite the prevalence of high-risk HPV genotypes (16, 18, 52), there was no statistically significant relationship between HPV subtype and NGAL levels (*p* = 0.29). This finding supports the idea that NGAL expressions are not directly influenced by viral genotype but rather by the downstream effects of HPV-driven carcinogenesis, such as persistent inflammation and local tissue remodeling. NGAL most likely reflects the tumor’s host interaction profile rather than viral properties, which can vary significantly even across high-risk HPV strains [1]. The relatively lower HPV positivity rate (63%) in our cohort compared with screening-based populations reflects the inclusion of biologically aggressive and HPV-independent cervical carcinomas, which are known to account for approximately 20–30% of invasive cervical cancers. These tumors are more frequent in advanced and inoperable disease and are characterized by strong inflammatory and stromal responses, which may further explain the strong association between NGAL and inoperability rather than HPV status.

NGAL levels in these HPV-negative cases were considerably greater than in healthy controls and like those in HPV-positive malignancies, with no subtype differences (*p* > 0.05). This suggests that NGAL increase is unrelated to viral oncogenesis and rather reflects tumor-associated inflammation and systemic load [15].

NGAL may thus function as a predictive biomarker in both HPV-related and non-HPV cervical malignancies, adding utility in tumors that lack the conventional viral driver but nevertheless demonstrate aggressive clinical behavior.

Traditional biomarkers like p16, Ki-67, and minichromosome maintenance (MCM) proteins are well-known immunohistochemical indications of HPV-induced oncogenesis and aberrant proliferation. Overexpression of p16 reflects E7-mediated inactivation of the retinoblastoma protein and is frequently employed as a surrogate marker for high-risk HPV activity, which is significantly correlated with precancerous and malignant transformation. Ki-67 is a nuclear proliferation marker that correlates with abnormal cell cycle activity. It is usually coupled with p16 in dual-stain testing to improve specificity. MCM proteins (e.g., MCM2, MCM7) operate as replication licensing factors, have been associated with dysplasia, and are being studied as diagnostic and prognostic indications. One common disadvantage of these markers is that they need invasive tissue sample, which limits their application in longitudinal surveillance.

In contrast, NGAL in this investigation was tested in serum using ELISA, which provided a non-invasive and repeatable assessment. NGAL levels were considerably greater in advanced FIGO stages (e.g., FIGO IIIA = 205 ng/mL vs. FIGO IA1 = 105 ng/mL) and in inoperable patients compared to operable patients after therapy (179 vs. 123 ng/mL; *p* = 0.02). There was no correlation found between NGAL and HPV subtype distribution, implying that NGAL reflects tumor burden and systemic inflammatory activity rather than viral genotype.

Unlike tissue-based markers such as p16 and Ki-67, which reflect HPV-driven cell cycle dysregulation, NGAL provides a serum-based measure of host inflammatory response and tumor stroma interaction, thereby offering complementary biological information. NGAL’s non-invasive nature and dynamic activity make it a possible predictive biomarker for advanced and inoperable cervical cancer, with extra utility for long-term follow-up.

### 4.6. HPV Genotype Distribution by Age Group

This study highlights the high prevalence of high-risk HPV subtypes, with HPV 16 being the most frequent (26.03%), followed by co-infection with HPV 16 and 18 (8.22%). No low-risk types were detected, likely due to the oncological profile of the population. Overall, 63.1% of individuals were HPV-positive. HR types (particularly 16, 31, 51/52, and 18) dominate across the Balkans, while LR types appear in screening/low-grade samples (not malignancies). In Serbia (Vojvodina) [24], LR-42 is widespread in screening (13% of all kinds), whereas LR-6/11 is not found in HSIL/ASCH. According to PLOS, LR-6/11 accounts for almost 90% of genital warts in Europe [25]. The presence of LR in screening cohorts varies, with LR-42 being particularly prevalent in the Italian dataset [26].

Age-stratified analysis showed the highest HPV positivity in women aged 51–60 (72.3%), followed by the 31–50 groups. A decline was observed in women over 60, possibly due to reduced immune response or lower testing rates. These findings suggest that persistent or reactivated infections are more common in midlife, supporting prior evidence on immunosenescence.

Although no direct link was found between HPV subtype and NGAL levels, NGAL remains a valuable marker of tumor aggressiveness, particularly in inoperable and squamous cell carcinoma cases. The combination of HPV profiling with NGAL and histopathology may improve cervical cancer risk assessment and clinical decision making [1].

To qualify as a biomarker, a molecule must be both specific and sensitive. In our study, NGAL meets specificity by being strongly associated with advanced FIGO stages, inoperability, and aggressive histology, but not with unrelated factors such as HPV subtype. It meets sensitivity by consistently reflecting biological differences between operable and inoperable cases, and by showing clear trends with disease progression, even when conventional parameters remain unchanged. Thus, NGAL demonstrates the key criteria required of a biomarker in cervical cancer, especially as a prognostic indicator of tumor aggressiveness and systemic inflammatory burden [27].

### 4.7. Strengths and Limitations

Its strengths include the incorporation of NGAL quantification with FIGO staging, operability status, and HPV genotyping, resulting in a unique serum-based perspective on cervical cancer biology.

This study’s single-center methodology and small sample size limit the generalizability of findings and exclude survival analysis.

An important limitation of this study is the absence of tissue-level NGAL quantification by qPCR or immunohistochemistry. Therefore, we cannot determine whether circulating NGAL originates predominantly from tumor cells, stromal components, or inflammatory infiltrates. However, this limitation is also consistent with our main finding that serum NGAL reflects systemic inflammatory and tumor-associated host response rather than tumor-specific molecular expression. Future studies combining serum NGAL with tissue-based NGAL expression are required to clarify its cellular source and biological significance.

NGAL may be clinically useful for follow-up of patients with cervical carcinoma particularly in those with locally advanced or inoperable disease undergoing radiotherapy or chemoradiotherapy, where it may reflect treatment-related biological response and tumor–host inflammatory interactions. However, NGAL lacks tumor specificity and is strongly influenced by systemic inflammation, infection, renal dysfunction, and metabolic or hepatic disorders. Therefore, NGAL should not be used for disease monitoring in patients with acute infections, chronic inflammatory conditions, or significant renal or hepatic impairment, as these conditions may lead to falsely elevated values unrelated to tumor activity. In this context, NGAL is best interpreted as a complementary biomarker within a clinically controlled setting rather than a stand-alone indicator of disease status.

## 5. Conclusions

This study shows that serum NGAL is not a reliable short-term indicator of treatment response in cervical cancer. However, NGAL levels were significantly higher in inoperable patients, indicating that NGAL primarily reflects tumor-associated systemic inflammatory burden and biological aggressiveness rather than HPV subtype or immediate therapeutic effects. The absence of any association between NGAL and HPV genotypes further supports its independence from viral subtype-specific oncogenic pathways.

These findings suggest that NGAL may have value as a serum-based biomarker for advanced and inoperable disease, rather than for early-stage or operable tumors. Nevertheless, the lack of long-term clinical outcome data limits definitive prognostic interpretation, and further validation in larger prospective cohorts with survival and recurrence endpoints is required.

Future validation should be performed in large, multicenter prospective cohorts with longitudinal follow-up to confirm the prognostic value of NGAL for disease aggressiveness, inoperability, and clinical outcomes. Integration of serum NGAL into existing clinical algorithms together with FIGO staging, histopathology, and established molecular and inflammatory biomarkers could enable more accurate risk stratification and support individualized therapeutic decision making in cervical cancer.

## Figures and Tables

**Table 1:** FIGO staging and NGAL results, the suffixes ‘c’ indicates clinical staging and refers to inoperable or non-surgically treated patients, CCRT—Concomitance Chemoradiotherapy (includes External an Internal radio treatment), BRT-Brachytherapy, SD—Standard deviation.

Classification	Patients [%]	Treatment (%)		NGAL [ng/mL]			
		CCRT	BRT	Pre	SD	Post	SD
Figo IA1	5	0	5	105	32.7	119	34.3
Figo IA2	9	2	7	98.4	35.4	98.8	38.1
Figo IB1	13	13	0	103	41.2	103	84.9
Figo IB2	14	14	0	108	58.8	90.8	19.8
Figo IB3	3	3	0	100	28.9	66.1	25.7
Figo IIA	4	4	0	66.0	72.2	76.6	46.3
Figo IIB	6	6	0	174	68.3	302	47.3
Figo IIB c	13	13	0	167	90.7	148	41.7
Figo IIIA	4	4	0	205	210	221	256
Figo IIIA c	13	13	0	136	65.4	189	120
Figo IIIB	2	2	0	156	33.4	115	51.1
Figo IIIB c	9	6	3	159	57.0	164	126
Figo IIIC	3	3	0	189	88.6	205	139
Figo IIIC c	2	2	0	232	47.4	180	33.3
Initial tumor size (cm)							
≤2	27	15	12	103	42.0	102	44.3
>2 and ≤4	17	17	0	112	57.3	133	34.8
>4	56	53	56	178	84.6	175	109
Clinical nodal involvement							
No	44	70.4	29.5	101	64.7	107	67.1
Yes	56	100	0	143	63.7	157	85.2

**Table 2:** NGAL of both inoperable and operable patients.

Nr	Operability Status	Patients Number	NGAL (ng/mL)				
			Pre	SD	Post	SD	*p*-Value
1	Inoperable	37	155	64.6	179	67.1	0.04
2	Operable	63	122	69.8	123	92.7	0.06

**Table 3:** The percentage of HPV subtypes. Within other high risks are (26, 31, 33, 35, 39, 45, 51, 52, 53, 56, 58, 59, 66, 67, 73, and 82), Low risks include (6, 11, and 44). HPV-positive indicates that the group includes all cases with detected positive HPV subtypes in the testing, without differentiation by individual HPV genotypes.

Nr	HPV Testing	Percentage (%)
1	HPV_Negative (all)	36.9
2	HPV_Positive (all)	63.0
3	HPV_Other_HighRisk	24.6
4	HPV_16	26.0
5	HPV_18	2.74
6	HPV_16 and 18	8.22

## Data Availability

All datasets, materials, and results generated or analyzed during the current study are fully presented within this manuscript.

## Abbreviations

The following abbreviations are used in this manuscript:

NGAL	Neutrophil Gelatinase-Associated Lipocalin
HPV	Human Papillomavirus
FIGO	The International Federation of Gynecology and Obstetrics
ELISA	Enzyme-linked Immunosorbent Assay
PCR	Polymerase Chain Reaction
EBRT	External Beam Radiation Therapy
CBC	Complete Blood Count
AST	Aspartate aminotransferase
ALT	Alanine Aminotransferase
CEA	Carcinoembryonic Antigen
LDH	Lactate Dehydrogenase
Ca++	Ionized Calcium
TNM	Tumor Nodes Metastases
MCM	Minichromosome Maintenance
Ki-67	Proliferation Index
